# 1748. Viral and Bacterial Etiology of Acute Gastroenteritis in Children < 5 Years Old at a Major Pediatric Referral Center Before and During the COVID-19 Pandemic

**DOI:** 10.1093/ofid/ofad500.1579

**Published:** 2023-11-27

**Authors:** Olla Hamdan, Tess Stopczynski, Justin Z Amarin, Yasmeen Z Qwaider, Haya Hayek, Laura S Stewart, Rendie McHenry, Andrew J Spieker, James Chappell, Mary Wikswo, Sara Mirza, Natasha B Halasa

**Affiliations:** Vanderbilt University Medical Center, Nashville, Tennessee; Vanderbilt University Medical Center, Nashville, Tennessee; Vanderbilt University Medical Center, Nashville, Tennessee; Vanderbilt University Medical Center, Nashville, Tennessee; Vanderbilt University Medical Center, Nashville, Tennessee; Vanderbilt University Medical Center, Nashville, Tennessee; Vanderbilt University Medical Center; Division of Pediatric Infectious Diseases, Nashville, Tennessee; Vanderbilt University Medical Center, Nashville, Tennessee; Vanderbilt University Medical Center, Nashville, Tennessee; Centers for Disease Control and Prevention, Atlanta, Georgia; Centers for Disease Control and Prevention, Atlanta, Georgia; Vanderbilt University Medical Center, Nashville, Tennessee

## Abstract

**Background:**

In the United States, acute gastroenteritis (AGE) accounts for 1.5 million office visits, 200,000 hospitalizations, and 300 deaths in children each year. The specific etiology of AGE often remains unknown due to limited testing, including before and during the coronavirus disease 2019 (COVID-19) pandemic.

**Methods:**

We analyzed data collected in the emergency department (ED) and inpatient (IP) settings at Monroe Carell Jr. Children's Hospital at Vanderbilt University Medical Center as part of the CDC New Vaccine Surveillance Network (NVSN); a prospective population-based AGE surveillance study. Our analysis included children < 5 years of age presenting with vomiting (≥ 1 episode within 24 hours) or diarrhea (≥ 3 episodes within 24 hours) within 10 days prior to presentation and had a research stool specimen tested using xTAG® Gastrointestinal Pathogen Panel (Luminex). Our analysis included children with either bacterial or viral detection. Clostridioides difficile in children < 2 years of age was considered a colonization.

**Results:**

A total of 1,078 children with AGE were enrolled in the ED or IP from 01/12/2016 to 01/31/2023 and had a stool sample tested. We excluded 35 children with viral and bacterial co-detection and those with parasite detection. Our final analysis included 1,043 children with either viral detection (*n*=336, 32%), bacterial detection (*n*=70, 7%), or no detection (*n*=637, 61%). Notably, the viral group was younger and more likely to present with vomiting compared to the bacterial group **(Table 1)**. The most frequently detected viral pathogen was norovirus, followed by rotavirus, and then adenovirus. Salmonella was the commonly detected bacterial enteropathogen, followed by Clostridioides difficile. Most cases occurred in the pre-pandemic period, with negligible AGE cases reported from April to December 2020. However, AGE cases re-emerged in 2021 (**Figure 1**). A higher proportion of bacterial pathogens were detected during the COVID-19 pandemic.

**Table 1.**
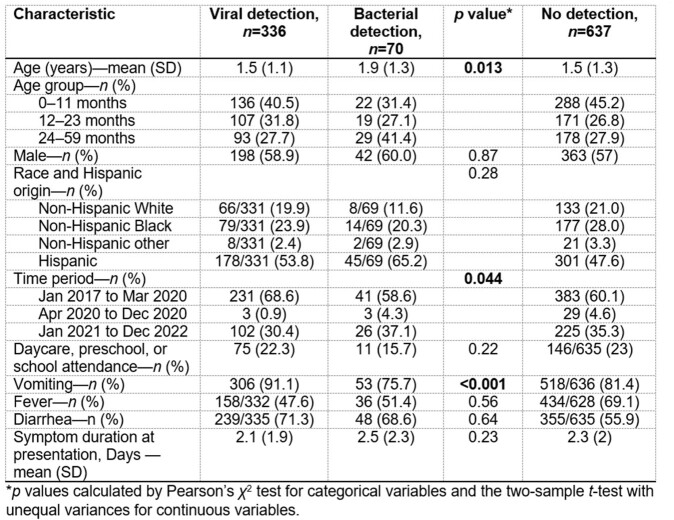
Demographic and clinical characteristics of children <5 years old who had a stool sample tested by xTAG® Gastrointestinal Pathogen Panel (N=1,043).

**Figure 1.**
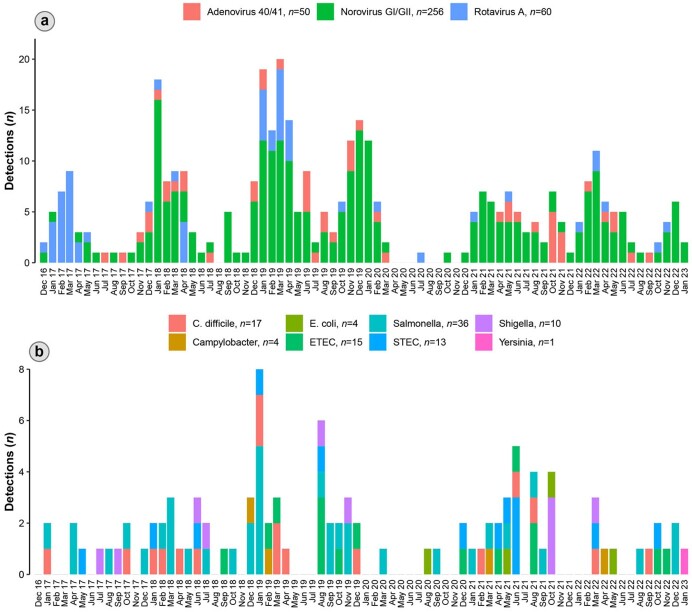
Monthly detection of viral (a) and bacterial (b) AGE pathogens from December 2016 to January 2023.

**Conclusion:**

Viral pathogens remained the predominant etiology of AGE in young children during the COVID-19 pandemic. Reduction of AGE cases likely coincided with stay-at-home recommendations utilized and less gathering opportunities during the COVID-19 pandemic.

**Disclosures:**

**Natasha B. Halasa, MD, MPH**, Merck: Grant/Research Support|Quidell: Grant/Research Support|Quidell: donation of kits|Sanofi: Grant/Research Support|Sanofi: vaccine support

